# Impact of the external knee flexion moment on patello-femoral loading derived from *in vivo* loads and kinematics

**DOI:** 10.3389/fbioe.2024.1473951

**Published:** 2025-01-15

**Authors:** Adam Trepczynski, Paul Kneifel, Mark Heyland, Marko Leskovar, Philippe Moewis, Philipp Damm, William R. Taylor, Stefan Zachow, Georg N. Duda

**Affiliations:** ^1^ Julius Wolff Institute, Berlin Institute of Health at Charité – Universitätsmedizin Berlin, Berlin, Germany; ^2^ Visual and Data-Centric Computing, Zuse Institute Berlin, Berlin, Germany; ^3^ Laboratory for Movement Biomechanics, ETH Zurich, Zürich, Switzerland

**Keywords:** patello-femoral force, external knee flexion moment, *in vivo* loading by telemetry, knee kinematics by mobile fluoroscopy, musculoskeletal modelling

## Abstract

**Introduction:**

Anterior knee pain and other patello-femoral (PF) complications frequently limit the success of total knee arthroplasty as the final treatment of end stage osteoarthritis. However, knowledge about the *in-vivo* loading conditions at the PF joint remains limited, as no direct measurements are available. We hypothesised that the external knee flexion moment (EFM) is highly predictive of the PF contact forces during activities with substantial flexion of the loaded knee.

**Materials and methods:**

Six patients (65–80 years, 67–101 kg) with total knee arthroplasty (TKA) performed two activities of daily living: sit-stand-sit and squat. Tibio-femoral (TF) contact forces were measured *in vivo* using instrumented tibial components, while synchronously internal TF and PF kinematics were captured with mobile fluoroscopy. The measurements were used to compute PF contact forces using patient specific musculoskeletal models. The relationship between the EFM and the PF contact force was quantified using linear regression.

**Results:**

Mean peak TF contact forces of 1.97–3.24 times body weight (BW) were found while peak PF forces reached 1.75 to 3.29 times body weight (BW). The peak EFM ranged from 3.2 to 5.9 %BW times body height, and was a good predictor of the PF contact force (*R* ^2^ = 0.95 and 0.88 for sit-stand-sit and squat, respectively).

**Discussion:**

The novel combination of *in vivo* TF contact forces and internal patellar kinematics enabled a reliable assessment of PF contact forces. The results of the regression analysis suggest that PF forces can be estimated based solely on the EFM from quantitative gait analysis. Our study also demonstrates the relevance of PF contact forces, which reach magnitudes similar to TF forces during activities of daily living.

## 1 Introduction

Total knee arthroplasty (TKA) is the ultimate treatment in late stage, severe knee osteoarthritis (OA). While TKA is among the most successful surgeries, it doesn’t restore function as consistently, as total hip replacement (Bourne et al., 2010), and up to 52% TKA patients have reported some degree of limitation in doing functional activities (Noble et al., 2005). Post TKA, patello-femoral (PF) complications have an incidence of up to 20% (Assiotis et al., 2019), and are one of the most frequent reasons for TKA revision (Lachiewicz and Soileau, 2006). Among these PF complications patella mal-tracking is among the most common conditions, and can lead to subluxation in 10% and dislocation in 2%–3% of TKA patients (Ritter et al., 1996), while an anterior knee pain incidence of 8% was reported (Sensi et al., 2011). Further PF complications include excessive polyethylene wear, or eventual implant loosening (D'Lima et al., 2003), which have been linked to PF contact loads (Han et al., 2007; Cho et al., 2011). Mechanical overloading of the PF joint can lead to patellar fractures, which are relatively rare with incidences of ∼1% (Choe et al., 2021), but can have dire consequences for the patients (Ortiguera and Berry, 2002; Sayeed et al., 2013; Choe et al., 2021; Masoni et al., 2023; Tsivelekas et al., 2024). Furthermore, patellar mal-tracking leading to excessive shear loading, and posterior tibial subluxation causing increased PF joint contact forces, are thought to play an important role in anterior knee pain (Petersen et al., 2014).

The PF joint is essential to the function of the knee, especially when the knee extensor mechanism has to balance high external flexion moments (EFM), for example, during squatting or getting up from a chair. So far, to the best of our knowledge, PF contact forces have not been measured *in vivo*. In the past, musculoskeletal modelling has been used to estimate the PF contact forces for different loading scenarios. Here, it has been shown that the magnitude of the PF contact forces can reach or even exceed the TF contact forces during activities of daily living (Trepczynski et al., 2012), generally occurring at higher joint flexion angles. However, modelling approaches show a high variability in predicted PF contact forces (Mason et al., 2008), which can be partially attributed to inter-patient differences such as anatomy, muscle activation patterns, speed of execution, or muscle strength. Due to the lack of direct *in vivo* measurements, it remains unclear how much of the reported variability in PF contact forces is due to different model assumptions, like the effective lever arm of the knee extensor mechanism, the ratio of patellar tendon to quadriceps force, or the level of antagonistic muscle co-contraction.

This study aims to determine PF contact forces based on *in vivo* measurements of internal knee kinematics and TF contact forces to verify whether the EFM is an effective predictor of PF contact force.

## 2 Materials and methods

### 2.1 Patients

The patients analysed in this investigation have been described in previous studies utilizing the CAMS-Knee Dataset (https://cams-knee.orthoload.com/) (Taylor et al., 2017; Trepczynski et al., 2019; Trepczynski et al., 2021; Heyland et al., 2023) and are also summarized in Table 1. The inclusion criteria for the instrumented TKA were: patients about to receive a TKA, at least 50 years of age, not more than 100 kg body mass, no other electronic implants (e.g., cardiac pacemakers), and sufficient fitness to participate in subsequent gait analyses. Six out of the nine patients originally implanted with the instrumented TKA, were able to participate in the CAMS-Knee measurements involving mobile fluoroscopy, that were performed 5–7 years postoperatively (Taylor et al., 2017). Due to the rarity of patients with instrumented knee implants, no additional selection criteria were imposed in the current study, and all six patients from the CAMS-Knee dataset were included. In this study, we focus on two activities known to produce large EFMs, sit-stand-sit and squat, of which each patient preformed 5–6 repetitions. Additionally to previously reported parameters such as the hip-knee-ankle (HKA) angle, we have now also quantified the patella height for the CAMS-Patients in terms of the Insall-Salvati-Index (ISI) (Insall and Salvati, 1971). We based the ISI on the patellar position reconstructed from fluoroscopy during the investigated activities, where we took the mean ISI value of all data points within 20°–70° knee flexion range for each patient (Phillips et al., 2010).

**TABLE 1:** The anthropometric patient data of at the time of the measurement, body-mass-index (BMI), Insall-Salvati-Index (ISI), hip-knee-ankle (HKA) angle.

Patient	Gender	Age [years]	Body height [cm]	Body mass [kg]	BMI [kg/m^2^]	ISI [-]	HKA angle [°]
K1L	m	70	175	101	33	1.24	3
K2L	m	78	169	91	32	1.33	5
K3R	m	77	173	100	34	1.48	3.5
K5R	m	65	174	96	32	1.21	1
K7L	f	80	165	67	24	0.87	6.5
K8L	m	76	175	79	26	1.35	4
MEAN		74.3	171.8	88.9	30.1	1.25	3.8

### 2.2 Reconstruction of patello-femoral kinematics

The CAMS-Knee dataset contains the reconstructed tibio-femoral (TF) kinematics based on the metal TKA components, which are clearly visible in the fluoroscopic images (Taylor et al., 2017). However, the dataset does not contain any reconstruction of the patellar kinematics, which was a key prerequisite for this study.

**FIGURE 1:**
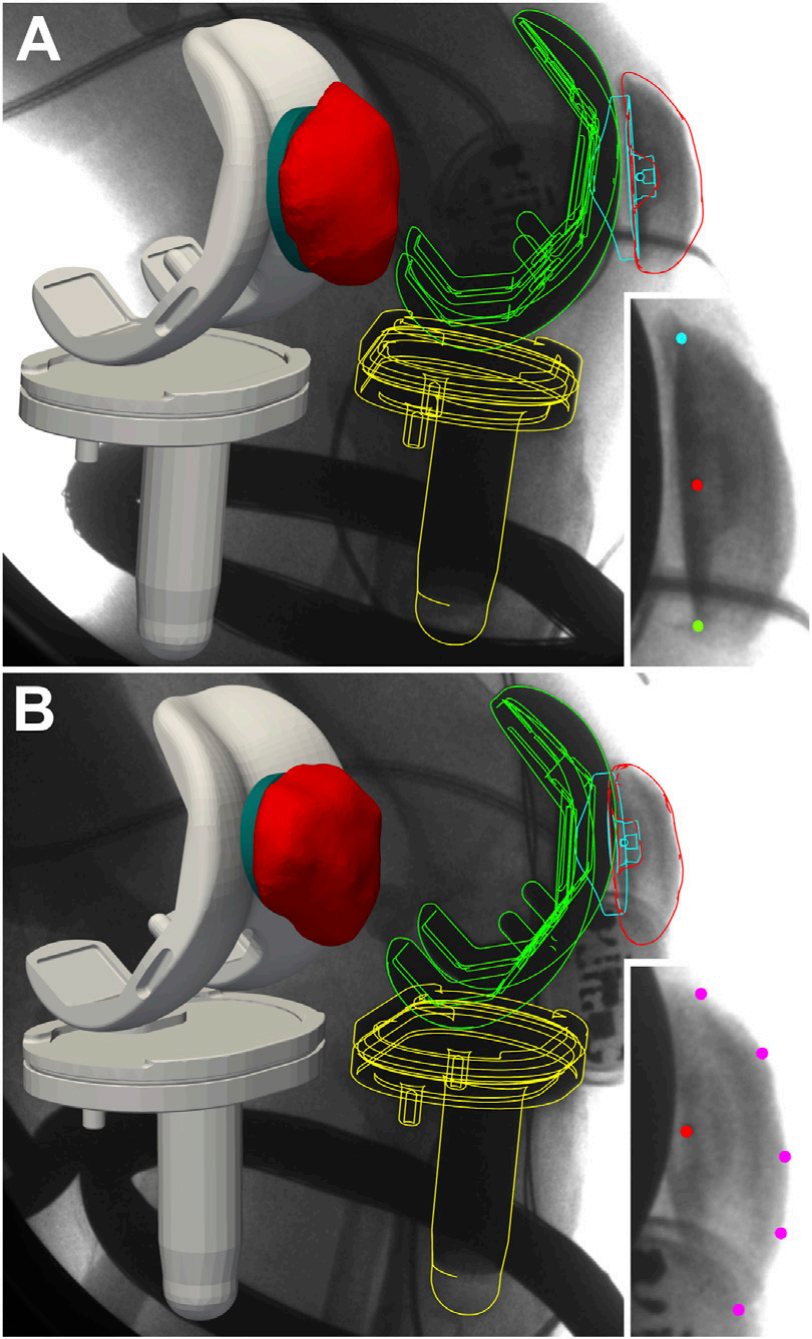
Reconstruction of patellar kinematics based on manually identified points in the fluoroscopic images (detail views in the bottom right corners) and automatic alignment with the femoral groove. The patella location in the image plane was determined based on the metal marker ball embedded in the patellar implant (red dot). For the patella flexion one of two methods was chosen, depending on the visibility of the patella: If the bone-implant interface was fully visible, its most proximal (cyan dot) and distal (green dot) ends were used **(A)**, otherwise the anterior boundary of the patella bone was marked with 5 points (purple dots) **(B)**.

Since the focus of the CAMS-Knee study was the accurate capture of the TF kinematics, the visibility of the patella and its characteristic radiographic features was limited in parts of the image data due to occlusions or overexposure. This limitation made it infeasible to reconstruct the patellar kinematics using the same approach that was used for the TF components (List et al., 2017). Instead, we developed a semi-automatic method based on patellar points identified manually on the 2D fluoroscopy images (in-plane kinematics), which were automatically combined with the 3D location of the femoral component (out-of-plane kinematics) using a pin-hole-camera projection model (Hartley and Zisserman, 2004), to yield the 3D location of the patella. For the alignment of the in-plane position, the usually clearly visible metallic marker ball at the centre of the retro-patellar polyethylene implant was used (Figure 1). The out-of-plane position was then adjusted by aligning the centre of the patella implant with the centre of the patellar groove of the femoral component, while preserving the location of the metal ball projected onto the image plane. The patellar tilt was adjusted to align the relatively congruent retro-patellar and femoral implant geometries. For the alignment of the in-plane rotation (patella flexion), one of the following two approaches was used, depending on the visibility of the patella. If the interface between the patellar bone and the retro-patellar implant was clearly visible, its most proximal and distal points were marked (Figure 1A), otherwise 5 points were placed on the anterior patellar bone boundary (Figure 1B). In both approaches, the patella flexion was adjusted to align the patellar bone boundaries projected onto the image plane with the corresponding points marked on the image. The placement of the retro-patellar points was performed by one person, while the anterior patellar points were placed by a separate investigator who also checked the retro-patellar points. The resulting PF kinematic degrees of freedom were plotted for each trial and checked visually for outliers and discontinuities, which were then corrected as necessary. The combined TF and PF implant kinematics were also rendered as 3D animations from different perspectives (sagittal, frontal), and checked visually.

During phases at which the metallic marker ball was not visible in the fluoroscopic images the patellar kinematics were interpolated by fitting curves into the reconstructed time points of a given patient and activity. The curve fitting was based on the relative patello-femoral position (in polar coordinates) and patella rotation in the sagittal plane of the femoral component (patella flexion), into which quadratic functions with respect to knee flexion were fitted. The fitted curves matched the reconstructed kinematic parameters with *R* ^2^ values of at least 0.98 and root mean square errors (RMSE) of 0.17–0.38 mm for the location’s radial coordinate, 0.33°–0.72° for the location’s angular coordinate, and 0.65°–1.66° for the patella flexion.

Of the 6569 fluoroscopic frames for which patella kinematics was reconstructed, 3740 were registered using the proximal/distal implant interface method (Figure 1A), and 1603 using the anterior boundary method (Figure 1B), while the remaining 1226 were interpolated using quadratic curve fitting.

### 2.3 Musculoskeletal modelling

The musculoskeletal model employed in this study was based on the previously described approach (Trepczynski et al., 2012), which was modified to incorporate the *in vivo* measured TF loads, as well as TF and PF kinematics from fluoroscopy. The model was constrained to track the measured TF force within 5% error as described in an earlier study (Trepczynski et al., 2018).

To reconstruct the skeletal kinematics of the lower limb, the 3D patient-specific anatomy from CT data, and skin marker-based kinematics were used as previously described (Trepczynski et al., 2012). However, in this study the skin marker-based kinematics were modified based on the in-plane TF kinematics from fluoroscopy. Unlike our previous studies, where the PF kinematics were based purely on geometric estimations, this study used *in vivo* PF kinematics directly reconstructed from fluoroscopy, as described in the previous section.

The effective lever arm of the knee extensor mechanism was derived from the fluoroscopic data, based on the functional knee flexion axis computed from TF kinematics (Taylor et al., 2010; Ehrig et al., 2011), and the patellar tendon force line of action computed from patello-tibial kinematics, which was applied to individual attachment sites identified from the image data (Kneifel et al., 2023).

**FIGURE 2:**
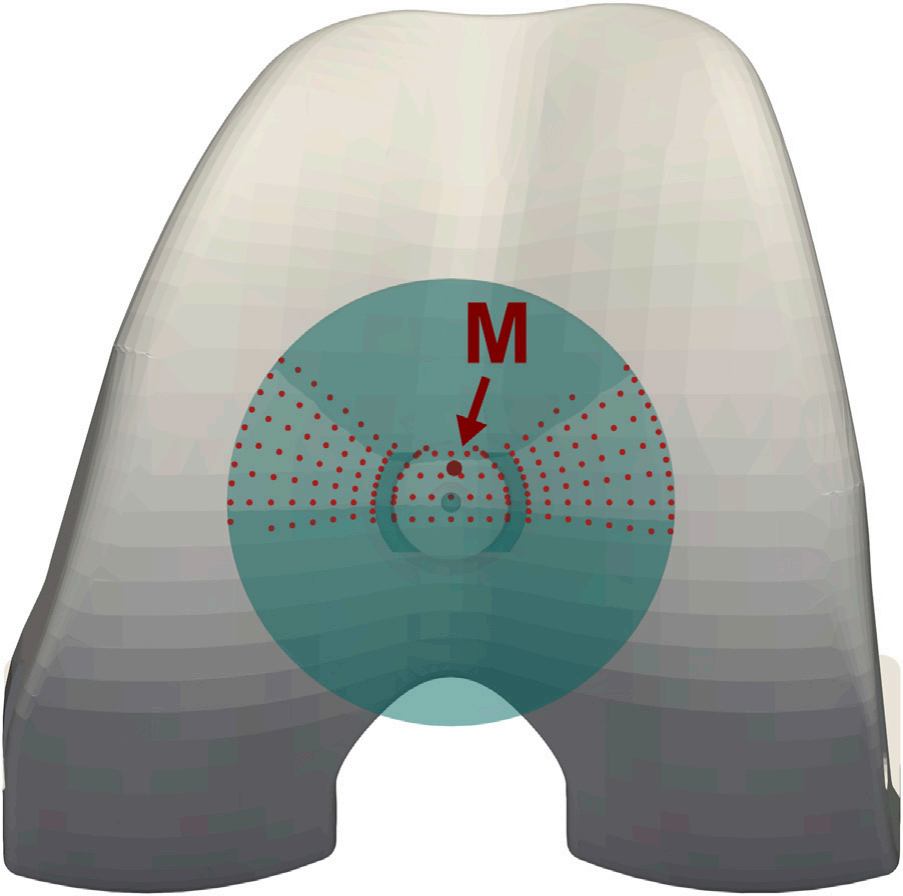
Estimation of the patello-femoral contact area, and of the effective point of application for the patello-femoral force (M), based on the patellar kinematics reconstructed from fluoroscopy.

The ratio of the patellar tendon force to quadriceps force (TQR) was determined based on an equilibrium condition for moments acting at the patella in the sagittal plane around the effective PF contact point. The effective PF contact point was estimated based on the fluoroscopic patella location relative to the femur, which was used to identify the approximate PF contact area: all nodes of the patellar implant surface that were closer than 1 mm to the femoral component were considered to be in physical contact, and their positions were averaged to yield the effective PF contact point (Figure 2). Subsequently, the PF contact force was computed from the patellar tendon and quadriceps forces, based on the assumption of equilibrium for these three forces acting on the patella.

### 2.4 Data processing and analysis

To make the loading comparable across patients, the forces were normalized to multiples of each individual’s body weight (BW), while the moments were normalized to body weight times body height (BWHt). In order to plot averaged loading profiles for all trials of each patient with respect to knee flexion, the data was transformed from the time domain to the flexion domain and sampled in 5° knee flexion steps, which was found to provide sufficient resolution to correctly represent the curves. The linear regression analyses were performed using the original data points. Since this study focuses on the loading of the knee extensor mechanism, only time points with clearly positive external flexion moments (EFM >0.001 BWHt) were considered in the linear regression analyses between the EFM and the contact force in the TF and PF joints.

The manual localization of patellar points in the fluoroscopic image data was performed in the ImageJ software (PointPicker-Plugin), while the automatic out-of-plane patella registration, PF contact point estimation, result processing, plotting and statistical analyses were performed using custom code written in R (R-Core-Team, 2022).

## 3 Results

### 3.1 Load related parameters

**FIGURE 3:**
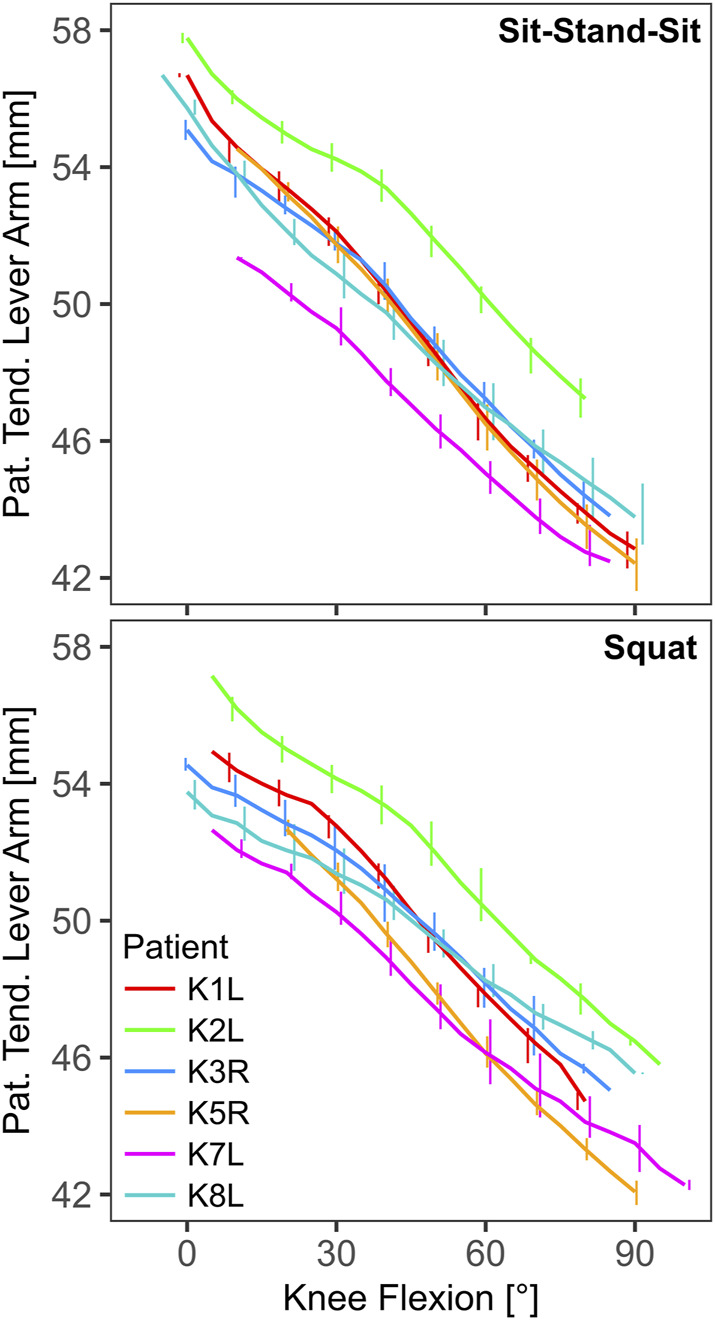
The effective lever arm of the patella tendon in the sagittal plane as function of knee flexion (line: mean, bars: range).

**FIGURE 4:**
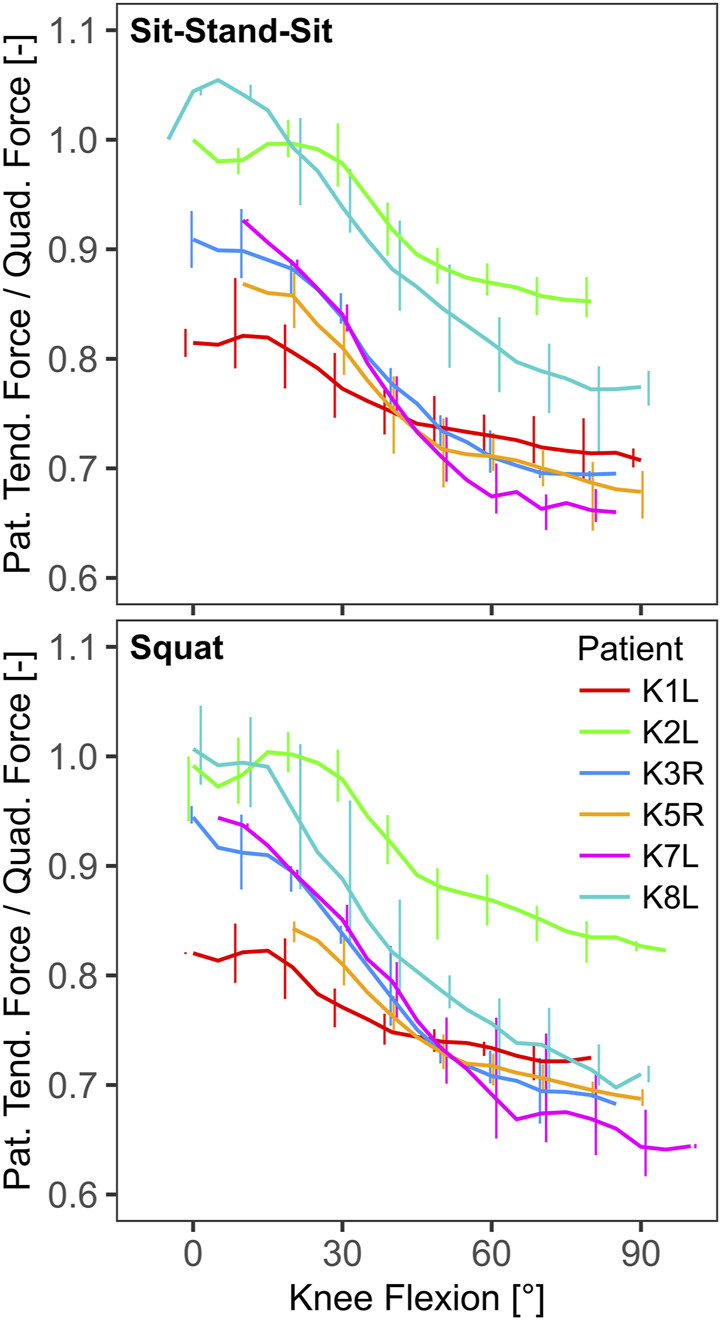
The ratio of patella tendon force to quadriceps force as function of knee flexion (line: mean, bars: range).

The mean peak knee flexion ranged from 77° for patient K1L during squat to 93° for K5R during both activities (Table 2). The effective lever arm of the patella tendon generally decreased with knee flexion, with mean peak values in extension ranging from 51 mm to 58 mm, and mean lowest values in flexion ranging from 42 mm to 47 mm (Table 2; Figure 3). The TQR was also highest in extension, with mean peak values ranging from 0.84 to 1.06 (Table 2). With increasing flexion the TQR decreased, with mean trial minima ranging from 0.61 to 0.83 (Table 2; Figure 4).

**TABLE 2:** Ranges and peak values for kinematic and loading parameters. F_TF_, tibio-femoral force; EFM, external flexion moment; F_PF_, patello-femoral force (mean ± standard deviation).

Acitivity	Patient	Max. Knee flexion [°]	Pat. Tend. Lever Arm [mm]	Pat. Tend. to quadriceps force Ratio [-]	Max. F_TF_ [BW]	Max. EFM [%BWHt]	Max. F_PF_ [BW]
Sit-Stand-Sit	K1L	90 ± 3	43 ± 1 – 56 ± 1	0.70 ± 0.01 – 0.85 ± 0.02	3.04 ± 0.25	5.1 ± 0.6	2.99 ± 0.31
	K2L	81 ± 2	47 ± 0 – 58 ± 0	0.83 ± 0.01 – 1.01 ± 0.01	2.41 ± 0.15	3.5 ± 0.2	1.89 ± 0.09
	K3R	88 ± 0	44 ± 0 – 55 ± 0	0.68 ± 0.00 – 0.95 ± 0.03	2.68 ± 0.06	5.0 ± 0.3	2.84 ± 0.12
	K5R	93 ± 2	42 ± 0 – 54 ± 1	0.65 ± 0.01 – 0.87 ± 0.03	3.17 ± 0.06	5.1 ± 0.2	2.99 ± 0.10
	K7L	88 ± 2	42 ± 0 – 51 ± 0	0.64 ± 0.01 – 0.92 ± 0.01	2.39 ± 0.07	3.6 ± 0.2	2.11 ± 0.08
	K8L	91 ± 0	43 ± 0 – 57 ± 0	0.72 ± 0.01 – 1.06 ± 0.00	3.07 ± 0.13	4.3 ± 0.1	2.28 ± 0.09
Squat	K1L	77 ± 4	45 ± 1 – 56 ± 1	0.70 ± 0.01 – 0.84 ± 0.01	2.50 ± 0.15	3.7 ± 0.6	2.07 ± 0.23
	K2L	85 ± 11	47 ± 2 – 58 ± 0	0.82 ± 0.02 – 1.02 ± 0.01	2.54 ± 0.35	3.7 ± 1.1	2.06 ± 0.50
	K3R	78 ± 5	46 ± 1 – 55 ± 0	0.67 ± 0.01 – 0.98 ± 0.05	1.97 ± 0.17	4.0 ± 0.5	2.01 ± 0.23
	K5R	93 ± 2	42 ± 0 – 53 ± 1	0.68 ± 0.01 – 0.86 ± 0.01	3.21 ± 0.09	5.9 ± 0.4	3.29 ± 0.12
	K7L	92 ± 8	43 ± 1 – 53 ± 0	0.61 ± 0.01 – 0.95 ± 0.00	2.13 ± 0.04	3.2 ± 0.7	1.75 ± 0.19
	K8L	88 ± 2	46 ± 0 – 54 ± 0	0.67 ± 0.02 – 1.04 ± 0.03	3.24 ± 0.15	3.7 ± 0.2	2.24 ± 0.13

### 3.2 Forces and moments

**FIGURE 5:**
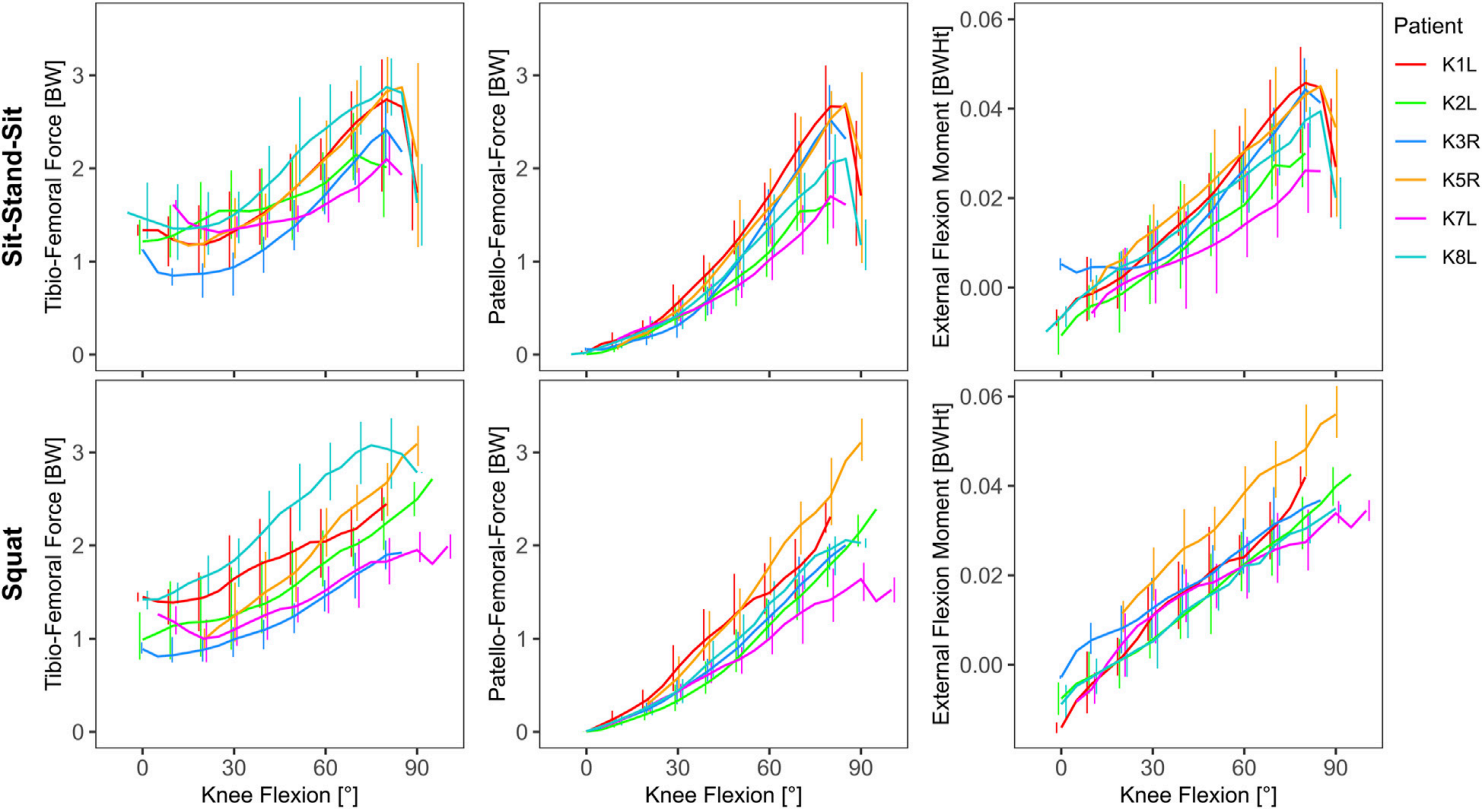
*in vivo* measured tibio-femoral force, calculated patello-femoral force and the external knee flexion moment as function of knee flexion (line: mean, bars: range).

The external and internal loading increased with flexion. The *in vivo* measured TF force was substantial even in extension, with mean minimal values ranging from 0.75 BW to 1.27 BW, rising with increasing flexion to mean peak values of 1.97 BW to 3.24 BW (Table 2). The highest absolute TF-force of 3220 N was observed in patient K1L during sit-stand-sit at 84° knee flexion. In extension, the external flexion moment (EFM) was close to zero or negative and reached mean peak values of 3.2 BWHt to 5.9 BWHt in flexion. The highest absolute EFM of 104 Nm was observed in patient K5R during a squat at 84° knee flexion (Table 2). The PF-force was near zero in extension and followed a similar profile with increasing flexion as the EFM, rising to mean peak values of 1.75 BW to 3.29 BW. The highest normalized PF-force of 3.40 BW was observed in patient K5R during a squat at 90° knee flexion, and was about 5% higher than the TF-force at the same instant. The highest absolute PF-force of 3302 N was observed in patient K1L during a squat at 84° knee flexion, and was about 8% higher than the concurrent TF-force (Table 2; Figure 5).

### 3.3 Relationships between loads

**FIGURE 6:**
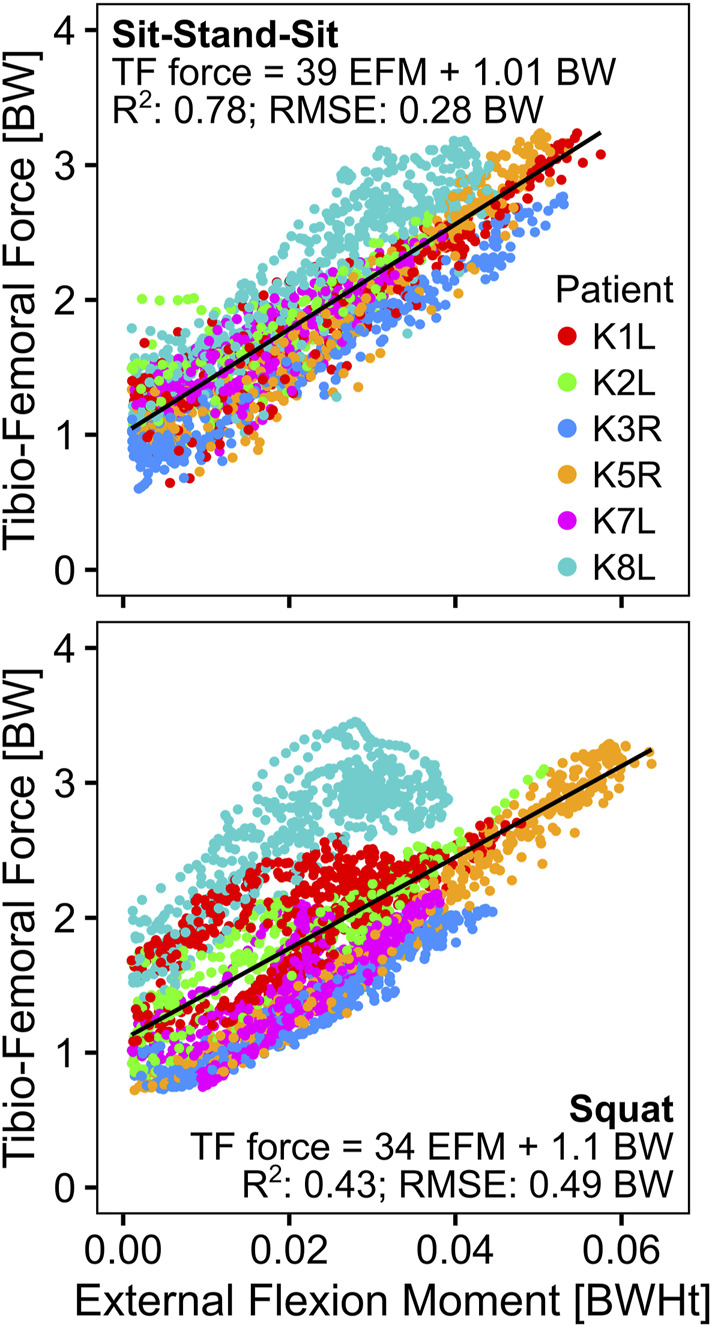
Relationship of the external knee flexion moment to the *in vivo* measured tibio-femoral force.

**FIGURE 7:**
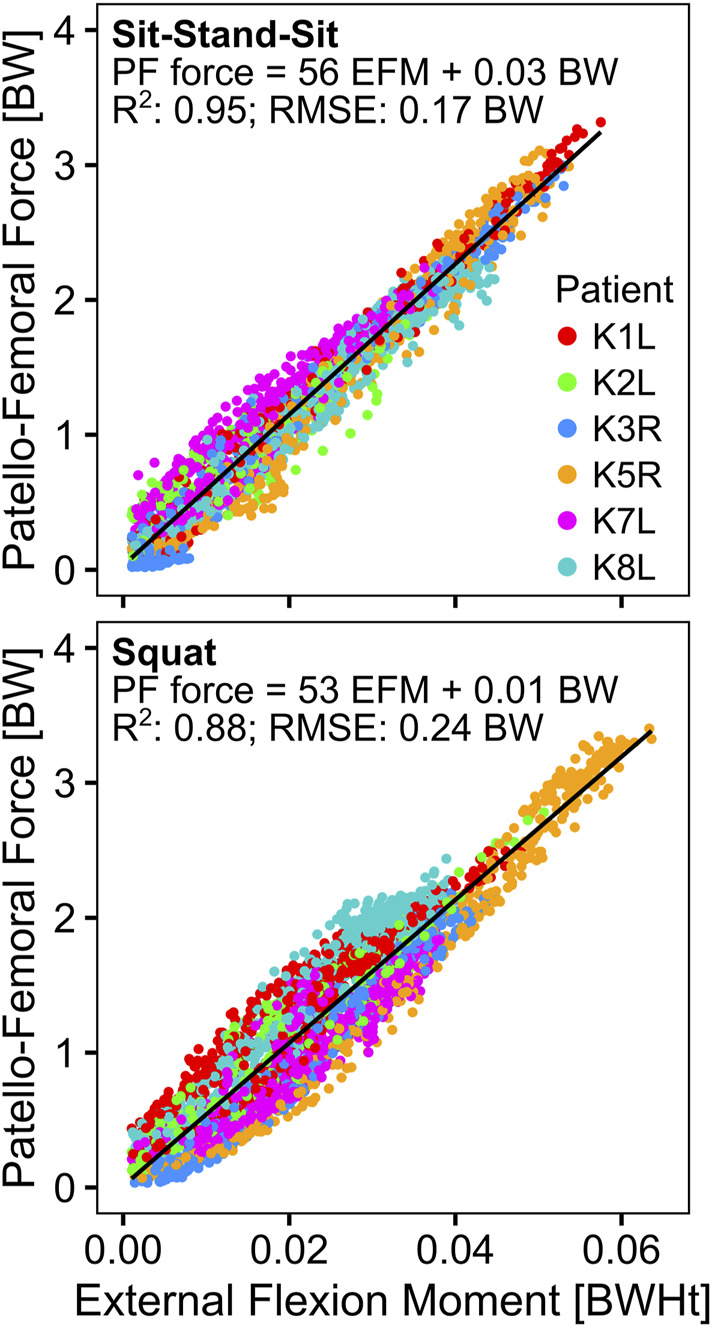
Relationship of the external knee flexion moment to the calculated patello-femoral force.

Linear regression of the EFM and the *in vivo* measured TF-force showed a good correlation for sit-stand-sit with an *R* ^2^ of 0.78, but a weaker one for squat with an *R* ^2^ of 0.43. For both activities, but especially for squat, patient K8L had exceptionally high TF-forces for a given EFM, reducing the overall correlation (Table 3; Figure 6). The correlation between the EFM and the PF-force was strong for both activities, with *R* ^2^ of 0.95 for sit-stand-sit and *R* ^2^ of 0.88 for squat. The intercept of the PF-force regressions was close to zero, allowing the regression coefficients to be interpreted as fixed ratios between PF and EFM (Table 3; Figure 7).

**TABLE 3:** The results of the linear regression analyses between the external flexion moment (EFM) in body weight * body height (BWHt) and the contact forces in body weight (BW), at the tibio-femoral (F_TF_) and the patello-femoral (F_PF_) joints. All correlations were significant with *p* < 0.001.

	F_TF_ [BW] = a [1/Ht] ⋅ EFM [BWHt] + b [BW]				F_PF_ [BW] = a [1/Ht] ⋅ EFM [BWHt] + b [BW]			
	*R* ^2^	RSME	a	b	*R* ^2^	RSME	a	b
sit-stand-sit	0.78	0.28	39	1.01	0.95	0.17	56	0.03
squat	0.43	0.49	34	1.10	0.88	0.24	53	0.01

## 4 Discussion

The knee extensor mechanism and the PF joint are crucial for lower limb function (Browne et al., 2005), and subject to high loading during activities with substantial flexion of the loaded knee, such as squatting and getting up from a chair (Mason et al., 2008). These activities are challenging for many TKA patients (Noble et al., 2005), but of high relevance for daily living. The high mechanical loading of the PF joint has been linked to various complications after TKA, such as anterior knee pain (Petersen et al., 2014), implant wear or loosening (D'Lima et al., 2003), and in some cases even implant failure or patellar fracture, often necessitating revision surgery (Choe et al., 2021). The key of rehabilitation is the right balance between improving function through training, while avoiding overloading of compromised structures which are still in the healing phase. This requires reliable knowledge of the internal loading in order to choose the right exercises (Song et al., 2023). However, so far, the individual forces at the patella during such activities have been estimated based on major assumptions about key parameters, and were therefore subject to substantial uncertainties.

For the first time, directly measured *in vivo* TF contact forces were combined with synchronous TF and PF kinematics captured by fluoroscopy, to determine PF contact forces during activities of daily living involving substantial knee flexion. This combination allowed to minimize the major uncertainties that are usually present in predictions of PF contact forces: The uncertainties regarding the effective lever arm of the knee extensor mechanism and the TQR were minimized by using fluoroscopy to determine the effective knee flexion axis, patella tendon force line of action and PF contact location. The uncertainty regarding the level of antagonistic muscle co-contraction was minimized by using telemetry to measure *in vivo* TF contact forces. Therefore, this study likely provides the most reliable estimation of PF contact forces so far, and can serve as a reference for biomechanical considerations in TKA design, implantation and rehabilitation.

Our hypothesis, that the EFM is highly predictive of the PF contact force was confirmed. The strong correlations between EFM and PF contact force, despite the wide range of patella heights, BMIs and frontal alignments of the patients included in the regression analysis, further suggests that the relationships reported here is generalizable to other subjects. The resulting coefficients of the regression thus allow to estimate PF forces based solely on the EFM using a simple formula (Table 3).

The PF contact force range found in this study during squatting at a knee flexion of 85° of 1.23–3.12 BW is consistent with the values from earlier studies in human subjects (Reilly and Martens, 1972; Dahlkvist et al., 1982; Komistek et al., 2005; Sharma et al., 2008), as summarized in a comprehensive review (Mason et al., 2008). The TQR determined in the current study was found to decrease with flexion, and be within ∼0.6–1.1, thus similar to TQR values from other studies (Mason et al., 2008).

The currently valid ISO-standard for testing the durability of the patellofemoral joint replacement prescribes simulating squatting with up to 120° of TF flexion and a PF contact force of up to 3 BW [ISO-14243-5: Sections 4 and 8.5, Equation 4; (ISO, 2019)]. The peak PF contact forces of over 3.2 BW found in our study at lower TF flexions suggest that the ISO-standard might not prescribe sufficiently high PF loading. While ISO-14243-5 cites the aforementioned review article (Mason et al., 2008), the recommended peak PF load for squatting seems to be based on two recent studies (Komistek et al., 2005; Sharma et al., 2008), while other studies in the review report PF loads of above 4 BW for 120° TF flexion (Reilly and Martens, 1972; Dahlkvist et al., 1982). A recent study, which aimed to provide more implant-specific testing recommendations and include patient variability, also reports peak PF contact loads of 4.6 BW, at ∼90° TF flexion during single-leg lunge (Navacchia et al., 2018). Considering the increasingly younger and more active TKA patient population, PF loads higher than observed in activities of daily living can be expected to occur more frequently.

By employing novel methodology, our study confirms that the PF joint experiences substantial contact forces, even during normal daily activities. For patients with smaller effective extensor lever arm, like K5R, the PF forces reach the level of the TF forces, while for those with greater extensor lever arm, like K2L, they stay ∼20% or ∼0.5 BW below the TF forces. Given similar peak load levels in the TF and PF joints, one would also expect similar sizes of the articulating areas in the natural knee. Here, it should be noted that the natural, non-pathological contact surfaces are more congruent in the PF joint than in the TF joint, which might allow the overall smaller PF joint to still have a comparable contact area to the TF joint. While quantifying these contact areas is beyond the scope of this study, the PF load levels provided here form a reliable basis for finite element analyses of the detailed contact mechanics of implants (Navacchia et al., 2018), as well as of the tissue level mechanobiology of the joint cartilage (Adouni et al., 2019; Faisal et al., 2019), and of the joint ligaments (Adouni et al., 2020). Extending our methodology towards such multiscale modelling approaches, could further improve the precision in identifying clinically relevant risks of overloading tissue and implants.

Despite involving an unprecedented number of patients with synchronous *in vivo* loads from telemetry and internal kinematics from fluoroscopy, our study has some limitations. The cohort was small, included only one woman, and was limited to one implant design, which should be considered when trying to generalize our results. While the maximal knee flexion achieved by our patients was substantial and covers the range most relevant for daily life, it does not include very deep flexion beyond 90°, where even higher PF loads are expected to occur. Furthermore, neither the friction at the PF contact nor the forces of the medial and lateral PF ligaments were considered in this study.

In conclusion, our results demonstrate the substantial loading of the PF joint, and provide a simple method to estimate PF contact forces based on the EFM from quantitative gait analysis. This can facilitate the investigation of PF loading as a possible source of anterior knee pain and functional limitations, as well as the evaluation of rehabilitation exercises in terms of potential PF overloading. This study therefore provides key information, which can contribute to improving the functional outcome of TKA in the future.

## Data Availability

The data analyzed in this study is subject to the following licenses/restrictions: Sample data that support the findings of this study is available on the CAMS-Knee-Project website. Restrictions apply to the availability of the implant geometry data, which were used under license for the current study, and so are not publicly available. Requests to access these datasets should be directed to https://cams-knee.orthoload.com/data/data-download.

## Abbreviations

BW, body weight; BWHt, body weight * body height; EFM, external (knee) flexion moment; HKA, hip-knee-ankle (angle); ISI, Insall-Salvati-Index; PF, patello-femoral; RMSE, root mean square errors; TF, tibio-femoral; TKA, total knee arthroplasty; TQR, ratio of the patellar tendon force to quadriceps force.
